# The Mycobacteriophage Ms6 LysB *N*-Terminus Displays Peptidoglycan Binding Affinity

**DOI:** 10.3390/v13071377

**Published:** 2021-07-15

**Authors:** Adriano M. Gigante, Francisco Olivença, Maria João Catalão, Paula Leandro, José Moniz-Pereira, Sérgio R. Filipe, Madalena Pimentel

**Affiliations:** 1 Research Institute for Medicines (iMed.ULisboa), Faculty of Pharmacy, Universidade de Lisboa, 1649-003 Lisboa, Portugal; amsgigante@gmail.com (A.M.G.); francisco.olivenca@ff.ulisboa.pt (F.O.); mjcatalao@ff.ulisboa.pt (M.J.C.); aleandro@ff.ulisboa.pt (P.L.); josemonizpereira@icloud.com (J.M.-P.); 2 UCIBIO-Applied Molecular Biosciences Unit, Departamento de Ciências da Vida, Faculdade de Ciências e Tecnologia, Universidade Nova de Lisboa, 2819-516 Caparica, Portugal; sfilipe@fct.unl.pt; 3 Laboratory of Bacterial Cell Surfaces and Pathogenesis, Instituto de Tecnologia Química e Biológica António Xavier, Universidade Nova de Lisboa, 2780-157 Oeiras, Portugal

**Keywords:** mycobacteriophage Ms6, LysB, phage lysis, mycobacteria, peptidoglycan binding

## Abstract

Double-stranded DNA bacteriophages end their lytic cycle by disrupting the host cell envelope, which allows the release of the virion progeny. Each phage must synthesize lysis proteins that target each cell barrier to phage release. In addition to holins, which permeabilize the cytoplasmic membrane, and endolysins, which disrupt the peptidoglycan (PG), mycobacteriophages synthesize a specific lysis protein, LysB, capable of detaching the outer membrane from the complex cell wall of mycobacteria. The family of LysB proteins is highly diverse, with many members presenting an extended *N*-terminus. The *N*-terminal region of mycobacteriophage Ms6 LysB shows structural similarity to the PG-binding domain (PGBD) of the φKZ endolysin. A fusion of this region with enhanced green fluorescent protein (Ms6LysBPGBD-EGFP) was shown to bind to *Mycobacterium smegmatis*, *Mycobacterium vaccae*, *Mycobacterium bovis* BGC and *Mycobacterium tuberculosis* H37Ra cells pretreated with SDS or Ms6 LysB. In pulldown assays, we demonstrate that Ms6 LysB and Ms6LysBPGBD-EGFP bind to purified peptidoglycan of *M. smegmatis*, *Escherichia coli*, *Pseudomonas aeruginosa* and *Bacillus subtilis*, demonstrating affinity to PG of the A1γ chemotype. An infection assay with an Ms6 mutant producing a truncated version of LysB lacking the first 90 amino acids resulted in an abrupt lysis. These results clearly demonstrate that the *N*-terminus of Ms6 LysB binds to the PG.

## 1. Introduction

Bacteriophages, also known simply as phages, are viruses that are able to propagate by infecting bacteria. The infection process used by double-stranded DNA (dsDNA) phages begins with the binding of the phage particle to a receptor present at the bacterial surface, followed by injection of the phage DNA into the bacterial host. Once in the cytoplasm, phages may undergo a lytic cycle and after replication of the phage DNA, and synthesis of the virion components, they are assembled to form new phage particles. In the last step, phages need to lyse the host to release the viral progeny that will start a new infection cycle [1]. To achieve this, phages need to overcome the barriers of the bacterial envelope. The lytic system employed by dsDNA phages depends on at least two proteins: an endolysin and a holin [1]. Holins are membrane proteins that permeabilize the cytoplasmic membrane (CM), determining the timing of lysis. Endolysins are enzymes that cleave covalent bonds in the peptidoglycan (PG) compromising the integrity of the cell wall [1,2]. Recent reports on phage-induced lysis have shown that phages that infect Gram-negative hosts require additional lysis proteins to compromise the last barrier to phage release, which is the outer membrane (OM) [3,4]. These proteins are named spanins and may exert their function as a complex formed by two spanin subunits, the i-spanin which integrates in the CM and the o-spanin, a lipoprotein that anchors to the OM, or as a unique protein, the u-spanin able to anchor to both membranes [2,5]. As a complex or as a sole protein, these proteins disrupt the OM, by a topological mechanism [6,7].

The best studied spanins are the Rz and Rz1 proteins of bacteriophage λ which are translated from two genes with a particular genetic architecture. *Rz1* is embedded in the +1 reading frame of *Rz* and codes for a lipoprotein, while *Rz* positioned downstream of *R* gene (encoding the endolysin) codes for an integral type II membrane protein. These two proteins are localized to the membranes during the morphogenesis period of an infection cycle, and interact with each other through their *C*-terminus forming a complex that spans the entire periplasm [6,8]. It has been proposed that phage-induced lysis in Gram-negative hosts is a three-step event in which holins accumulated in the CM form holes at a genetically defined time, permeabilizing the membrane, which consequently allows the endolysin to cleave specific bonds in the PG. Disruption of the PG meshwork induces conformational changes in the spanins, which brings the two membranes into close proximity resulting in the fusion of the CM and OM [4,6,7,9,10], eliminating the last barrier of the cell envelope.

Phages that infect mycobacteria, as dsDNA phages, also synthesize holins and endolysins to compromise the cytoplasmic membrane and the peptidoglycan layer respectively [11,12]. However, mycobacteriophages have to face a more complex cell envelope, where the presence of an outer membrane also constitutes a third barrier in the lysis process. The composition of the mycobacteria OM is completely different from the OM of Gram-negative bacteria. It is a bilayer with an inner leaflet mainly composed by mycolic acids, and an outer leaflet rich in several free (non-covalently bound) lipids (glycolipids, phospholipids and species-specific lipids) [13,14,15]. This OM or mycomembrane is covalently linked, through the mycolic acids to arabinogalactan, which in turn is covalently linked to peptidoglycan via an arabinogalactan network [16].

Today, more than 2000 mycobacteriophages have been sequenced [17], and for most of them, a third lysis gene, named *lysB*, has been identified in their lysis cassette. The work of Gil et al. [18,19] and Payne et al. [20] have shown that LysB proteins produced by phage Ms6 and Giles are lipolytic enzymes that cleave the ester bond that link the OM to the PG-AG complex. This suggests that these phages also lyse their hosts in three steps. Studies about lysis induced by mycobacteriophages are scarce, being the existent ones centralized in mycobacteriophages Ms6, Giles and D29 [11,20].

Like the majority of mycobacteriophage *lysB* genes, the Ms6 *lysB* is located downstream of *lysA* (endolysin); however, a multiple sequence alignment of LysB proteins shows that they are highly diverse, with some members showing <20% identity to each other. However, all have in common with the Ms6 LysB the motif G-X-S-X-G, characteristic of lipolytic enzymes [18,20].

We have previously reported the lipolytic activity of Ms6 LysB [18] and identified the mycobacteria OM as its main target since it cleaves the ester bond between the mycolic acids and arabinogalactan [19]. Recently, we showed that Ms6 LysB is essential for an efficient release of the new progeny virions at the end of a lytic cycle [21]. Using a Ms6 mutant lacking the *lysB* gene, infected cells did not undergo an explosive lysis as observed for the wild-type phage. In the absence of LysB, Ms6 particles remain trapped inside incomplete lysed cells, which results in a non-profit infection cycle.

A bioinformatic analysis has predicted, for several mycobacteriophage LysB proteins, the existence of an extended *N*-terminus harboring a peptidoglycan binding domain [20,22]. In the present work, we investigate the relevance of the *N-*terminal region of Ms6 LysB to the lysis process and experimentally determined the ability to bind peptidoglycan. In view of the current knowledge of phage-induced lysis by bacteriophage λ, we discuss the role of the Ms6 LysB peptidoglycan binding domain in the context of mycobacteria lysis.

## 2. Materials and Methods

### 2.1. Bacterial Strains, Plasmids, Bacteriophages and Culture Conditions

Bacterial strains, plasmids and bacteriophages used in this study are listed in Table 1. *E. coli*, *Staphylococcus aureus*, *Streptococcus pneumoniae*, *Bacillus subtilis and Pseudomonas aeruginosa* were grown at 37 °C in Luria-Bertani (LB) broth or agar when appropriate. For the growth of *E. coli* recombinant strains, media were supplemented with 100 µg/mL ampicillin. *M. smegmatis* mc^2^*155*, *M. tuberculosis* H37Ra, *M. vaccae* and *M. bovis* BCG were grown at 37 °C in Middlebrook 7H9 or 7H10 (BD Biosciences, San Jose, CA, USA) supplemented with 0.5% (*w/v*) glucose for *M. smegmatis* and *M. vaccae* or OADC (BD Biosciences, San Jose, CA, USA)) and 0.05% (*v/v*) Tween 80 for *M. tuberculosis* and *M. bovis* BCG. For the growth of *M. smegmatis* recombineering strain carrying pJV53 [23], media was supplemented with 15 μg/mL kanamycin.

### 2.2. DNA Manipulation and Plasmid Construction

DNA fragments were amplified by PCR using Pfu high-fidelity polymerase (Promega^®^, Madison, WI, USA). All oligonucleotides, listed in Table S1, were purchased from Thermo Scientific (Waltham, MA, USA) and were designed in order to have the restriction sites that allow the correct insertion into the vector. Restriction enzymes and T4 DNA ligase were purchased from ThermoScientific (Waltham, MA, USA) and New England Biolabs (Ipswich, MA, USA), respectively, and were used in accordance to the manufacturers’ instructions. DNA amplification, plasmid isolation, *E. coli* transformation and electrophoresis were carried out using standard techniques [28]. To construct plasmid pQE30:*egfp*, a DNA fragment containing the *egfp* gene was amplified from pEGFP-N1(Clontech Laboratories, Inc., Mountain View, CA, USA) using primers PrEGFPFw and PrEGFPRv and inserted into the KpnI and HindIII restrictions sites of vector pQE30 (Qiagen, Hilden, Germany), which allows the fusion of EGFP with a His_6_ tag at the *N*-terminus. Plasmid pQE30:*lysB*PGBD-*egfp* was obtained by amplification of the coding sequence of the first 90 amino acids (the putative PGBD) of Ms6 LysB with primers PrPGBDFw and PrPGBDRv using Ms6 genomic DNA as template. The PCR product was inserted into the BamHI and KpnI restriction sites of the previously constructed plasmid pQE30:*egfp*, allowing the production of the fusion protein Ms6LysBPGBD-EGFP carrying a *N*-terminal His_6_ tag. All constructs used in this study were verified and validated by nucleotide sequencing.

### 2.3. Protein Expression and Purification

Expression of the recombinant His_6_-LysB, His_6_-LysBPGBD-EGFP or His_6_-EGFP was induced from *E. coli* cells containing plasmids pMP302, pQE30:*lysB*PGBD-*egfp* or pQE30:*egfp*, respectively, with 1 mM IPTG for 4 h. Bacterial cells were then harvested by centrifugation, washed, resuspended in lysis buffer (50 mM NaH_2_PO_4_, 300mM NaCl, 10 mM Imidazole, pH 8.0) supplemented with a cocktail of protease inhibitors (Calbiochem, San Diego, CA, USA), and disrupted by passage through a French pressure cell press. Cell debris were removed by centrifugation, and the recombinant proteins were purified in an AktaPrime Plus^®^ system (GE Healthcare Life Sciences, Chicago, IL, USA) using a HisTrap FF Crude 1 mL Column (GE Healthcare Life Sciences, Chicago, IL, USA), according to the manufacturers’ instructions. The eluted protein fractions were dialyzed and stored in PBS at 4 °C, and concentrations were determined by the Bradford method, using bovine serum albumin (BSA) (New England Biolabs, Ipswich, MA, USA) as a standard.

### 2.4. Cells Binding and Fluorescence Assay

To test the ability of the recombinant proteins to bind to the bacterial cell surface, *M. smegmatis*, *M. tuberculosis* H37Ra, *M. vaccae* and *M. bovis* BCG cells were grown until an optical density at 600 nm (OD_600_) of 0.8, harvested by centrifugation and washed in SDS 1% in PBS for 1 h, with shaking at room temperature. Cells were then harvested by centrifugation and washed twice with PBS and incubated with 0.3 mg/mL of Ms6LysBPGBD-EGFP or EGFP proteins, for 45 min with shaking at room temperature. Cells were again harvested by centrifugation, washed trice with PBS, and 3 µL were placed on a 1% agar PBS microscope slide. In a parallel assay, *M. smegmatis* cells were pretreated with 0.3 mg/mL of Ms6 LysB (instead of SDS) for 45 min, with shaking at room temperature, followed by the washing steps describe above. Samples were observed using a Zeiss Axio ObserverZ1 microscope (Zeiss, Oberkochen, Germany) equipped with a Photometrics CoolSNAP HQ2 camera (Roper Scientific, Acton, MA, USA) using Metamorph software, Meta Imaging series 7.5 (Molecular Devices, San José, CA, USA) and analyzed using ImageJ software [29].

### 2.5. Preparation and Purification of Peptidoglycan (PG)

*S. aureus*, *B. subtilis*, *S. pneumoniae*, *E. coli* and *P. aeruginosa* CW and PG preparation were obtained as described in Filipe et al. [30], with modifications. Briefly, an exponential culture of the strain of interest was rapidly cooled in an ice/ethanol bath and harvested by centrifugation (13,000× *g*, 15 min, 4 °C). The pellet was resuspended in cold ultrapure water and boiled for 30 min with 4% (*w*/*v*) SDS. SDS was washed off by centrifugation in fresh warm Milli-Q water until no SDS could be detected by the Hayashi method [31].

The SDS-free pellets from *S. aureus*, *B. subtilis*, *S. pneumoniae* were broken in a FastPrep-24 5G (MP biomedicals, Irvine, CA, USA) homogenizer with acid washed glass beads (≤106 μm, Sigma-Aldrich, St. Louis, MO, USA). The samples from *S. aureus*, *B. subtilis*, *S. pneumoniae*, *E. coli* and *S. pneumoniae* were treated with DNase (10 μg/mL, Sigma-Aldrich, St. Louis, MO, USA) and RNase (50 μg/mL, Sigma-Aldrich, St. Louis, MO, USA), in 50 mM Tris-HCl pH 7.5, 20 mM MgSO_4_, and with Trypsin (100 μL/mL, Sigma-Aldrich, St. Louis, MO, USA) in 50 mM Tris-HCl pH 7.5, 20 mM MgSO_4_ and 10 mM CaCl_2_. These enzymes were inactivated by boiling in 1% (*w*/*v*) SDS and the clarified sample was washed twice with Milli-Q water, once with 8 M LiCl (VWR, Radnor, PA, USA), once with 100 mM ethylenediamine tetraacetic acid (EDTA, VWR, Radnor, PA, USA) pH 7.0 and once with acetone (Sigma-Aldrich, St. Louis, MO, USA) to remove SDS. The resulting purified PG from *E. coli* and *P. aeruginosa* was lyophilized and quantified by weight. The resulting sample from *S. aureus*, *B. subtilis* and *S. pneumoniae* was lyophilized and pure PG samples were obtained by removing the WTA by incubating CW in 48% (*v*/*v*) hydrofluoric acid (HF, Sigma-Aldrich, St. Louis, MO, USA) at 4 °C for 48 h. HF was washed off by centrifugation with 100 mM Tris-HCl pH 7.0. The resulting purified PG was lyophilized and quantified by weight.

To obtain PG from *M. smegmatis*, the mAGP was first purified accordingly to Bhamidi et al. [32]. To hydrolyze the mycolic acids, the mAGP was resuspended in 0.5% KOH in methanol and stirred at 37 °C for 4 days. The mixture was centrifuged, and the pellet was washed twice with methanol and twice with diethyl ether and dried by lyophilization. The resulting arabinogalactan-PG was digested with 0.05 N H_2_SO_4_ at 37 °C for 5 days to remove the arabinogalactan. The resulting insoluble PG was washed four times by centrifugation with water and dried.

Purity of PG was confirmed by high performance anion-exchange chromatography with pulsed amperometric detection (HPAEC-PAD) as described in Vaz et al. [33].

### 2.6. Peptidoglycan Binding Assays

Amounts of 90 µg of each purified protein and 25 µg of BSA (New England Biolabs, Ipswich, MA, USA) were added to 200 µg of peptidoglycan resuspended in a final volume of 300 µL of PBS, and incubated at room temperature with shaking, for 45 min. The supernatant was recovered by centrifugation at 350× *g* for 10 min, and the pellet was washed and resuspended in 500 µL of PBS and centrifuged at 2000× *g* for 5 min, washed again and centrifuged at 5500× *g* for 2 min. Both supernatant and pellet were analyzed by SDS-PAGE followed by Coomassie-blue staining and imaged using a ChemiDoc (Bio-Rad^®^, Hercules, CA, USA). Band intensities were quantified by densitometry using ImageJ2 software to compare the density of each band. The percentage of bound protein was calculated using the estimated amount of protein in the pellet fraction (bound) divided by the sum of the estimated protein amount on the pellet (bound) and supernatant (unbound) fractions. BSA was used as a loading-control (2.5 µg/lane) of all samples and the corresponding gel band used as a reference for density proportion calculations.

### 2.7. Construction of Ms6lysB∆PGBD

Deletion of 270 bp at the 5′ end of Ms6 *lys*B gene, corresponding to the putative PGBD was performed using the BRED technique in *M. smegmatis* as described previously [34]. Briefly, a 200-bp dsDNA was constructed from a 100-base oligonucleotide (*lysB*∆PGBD) with 50 bases of homology upstream and downstream of the deleted region, which was extended by PCR using two 75-base flanking primers PrExt∆lysBFw/PrExtlysB∆PGBDRv, each complementary to 25 bases at each end of the 100-mer. After purification the 200 bp fragment was co-electroporated with 200 ng of the wild-type Ms6 (Ms6*wt*) DNA into electrocompetent recombineering *M. smegmatis* mc^2^155:pJV53 as described previously [35]. Screening of phage plaques for selection of the mutant phage was performed by PCR with primers PrlysA120Fw/PrlysBendRv flanking the deleted region.

### 2.8. One-Step Growth Experiments

One-step growth curve assays were performed as described previously [35]. Briefly, 10^8^ phages were allowed to adsorb to 10^8^ *M. smegmatis* cells for 50 min at 37 °C. Non-adsorbed phages were then inactivated with 100 μL of 0.4% H_2_SO_4_ for 5 min followed by neutralization with 100 μL of 0.4% NaOH. The mixture was diluted 1:100 in 7H9 media and aliquots were taken at intervals of 30 min to quantify the number of phage particles. The obtained results are means of three independent experiments.

## 3. Results

### 3.1. Sequence Analysis of Ms6 LysB Reveals a Putative PGBD

**Figure 1:**
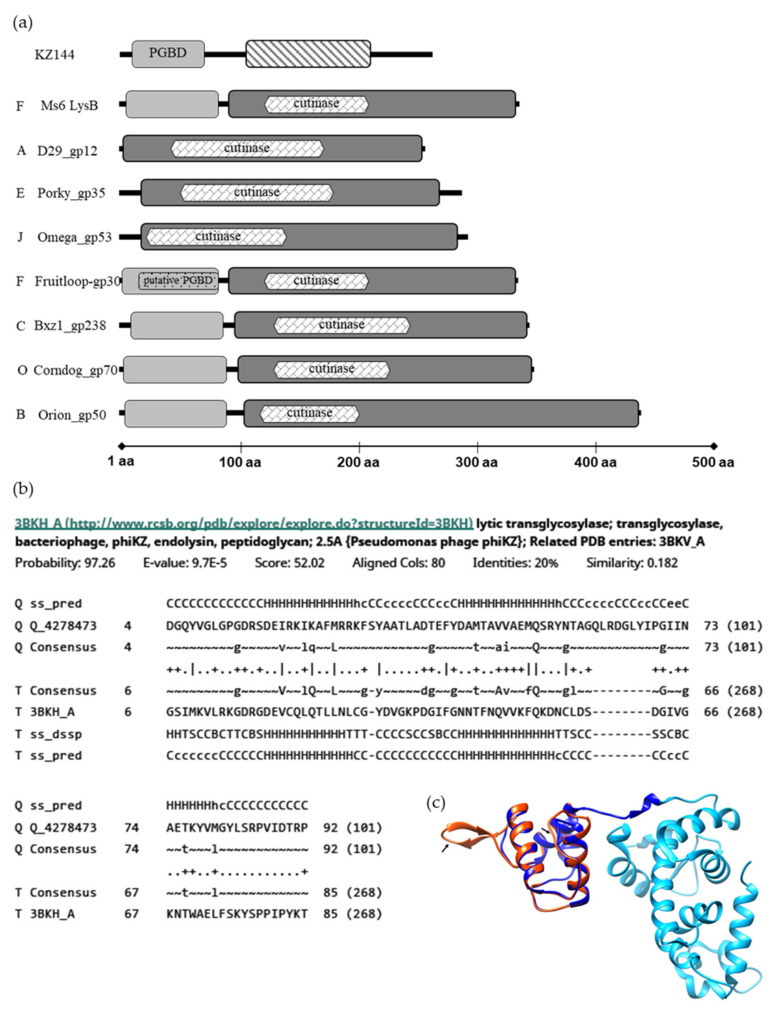
In silico analysis of LysB proteins. (**a**) Modular structure of KZ144 and representative mycobacteriophage’s LysB proteins. Regions with structural similarity to the KZ144 PGBD are shown in light grey blocks and a predicted catalytic region similar to D29 LysB in dark grey. φKZ gp144 lytic transglycosylase domain is marked with a strip pattern. The predicted PGBD in Fruitloop_gp30 is marked with a dot pattern. Each phage cluster is indicated by the letters on the left. The data shown are the result of a HHpred analysis for each depicted mycobacteriophage LysB. Predicted cutinase domains with MOTIF Finder are indicated within the LysBs catalytic region. (**b**) Alignment between the *N*-terminus of Ms6 LysB and the PGBD of φKZ endolysin as obtained from HHPred. (**c**) Structural model of Ms6 LysB using KZ144 (PDB ID-3BKH) as the template obtained from the SWISS-MODEL server. Catalytic and *N*-terminal domains of KZ144 are represented in light and dark blue, respectively. The generated structure of the *N*-terminal sequence of Ms6 LysB is represented in red. The sequences with lower similarity with target (low scored) are indicated by an arrow. Image was created with UCSF Chimera [44] and by superposing the PDB structure 3BKH and the constructed Ms6 LysB model.

Previous studies have shown that LysB homologues encoded by mycobacteriophages belonging to diverse clusters, although sharing the conserved pentapeptide G-X-S-X-G, characteristic of enzymes with lipolytic activity, are highly diverse [20,22]. Protein length may vary between 244 and 458 amino acids, and many LysB proteins have an extra *N*-terminus, 73–106 residues in length, that is absent in several other LysB proteins (Figure 1a). This diversity also extends to the structural level, with 32 different types of folds having been identified in a CATH analysis for 72 LysB proteins [22].

Analysis of Ms6 LysB by HHPred [36,37] predicts two regions, one starting at amino acid 91, containing the catalytic domain, showing high similarity with the structure of mycobacteriophage D29 LysB (3HC7_A) amino acids 1–253, with an e-value of 1.2 × 10^−39^. This region includes a predicted cutinase motif (120–208) (pfam01083) identified through the MotifFinder tool [38], which is in agreement with the previously identified lipolytic activity of this protein [18]. Residues 1–90 are part of the mentioned extra *N*-terminus. Although for some LysB homologues, a peptidoglycan-binding motif (PF01471) was predicted within this region (Fruitloop_30, JAMaL_44, Che9c_26, TM4_30) [22,39], no such motif was predicted for the Ms6 LysB. Of note is the fact that Fruitloop_30 shares with the amino acid sequence of Ms6 LysB an identity of 98% and has the same CATH domain (2mprA00) [22].

The only experimentally determined structure of a LysB protein is that of mycobacteriophage D29, and unfortunately, this shorter protein lacks this extra *N-*terminus, and consequently there is no structural information available for this region. Interestingly, inspection of the Ms6 LysB *N*-terminus (not present in D29 LysB) showed structural similarity with the peptidoglycan-binding domain (PGBD) of the *Pseudomonas aeruginosa* bacteriophage φKZ endolysin (KZ144) 3BKH_A, with an e-value of 9.7 × 10^−5^ (Figure 1b).

KZ144 is a lytic transglycosylase containing a PGBD (Pfam01471) at the *N*-terminus for which the peptidoglycan binding capacity has been experimentally demonstrated [40]. 

By performing homology modelling (SWISS-MODEL web-server, https://swissmodel.expasy.org/, accessed on 25 June 2021) [41,42,43], using the available structure of KZ144 (PDB ID 3BKH) as the template and the *N*-terminal sequence of Ms6 LysB as the target, a 3D structure model was generated (Figure 1c). The structural alignment of the obtained Ms6 LysB model and KZ144 shows that three secondary structural elements (α-helices) within the *N*-terminal sequence of Ms6 LysB presented a high predicted local similarity to target (<0.6). Two regions were found with a low score (predicted local similarity to target >0.6), namely, the RKFSYAA sequence between α-helices 1 and 2 and the TAGQLRDGLYI sequence between α-helices 2 and 3, for which a β-strand was assigned. It is noteworthy that a Blastp search identified a peptidoglycan binding protein of *Mycobacterium fortuitum* (WP_064866887) with similarity to Ms6 LysB (49% sequence identity). Taking into consideration all these observations, it is tempting to hypothesize that the *N*-terminus of Ms6 LysB encompasses a peptidoglycan binding domain.

### 3.2. The Ms6 LysB N-Terminus Binds to Mycobacterial Cells

**Figure 2:**
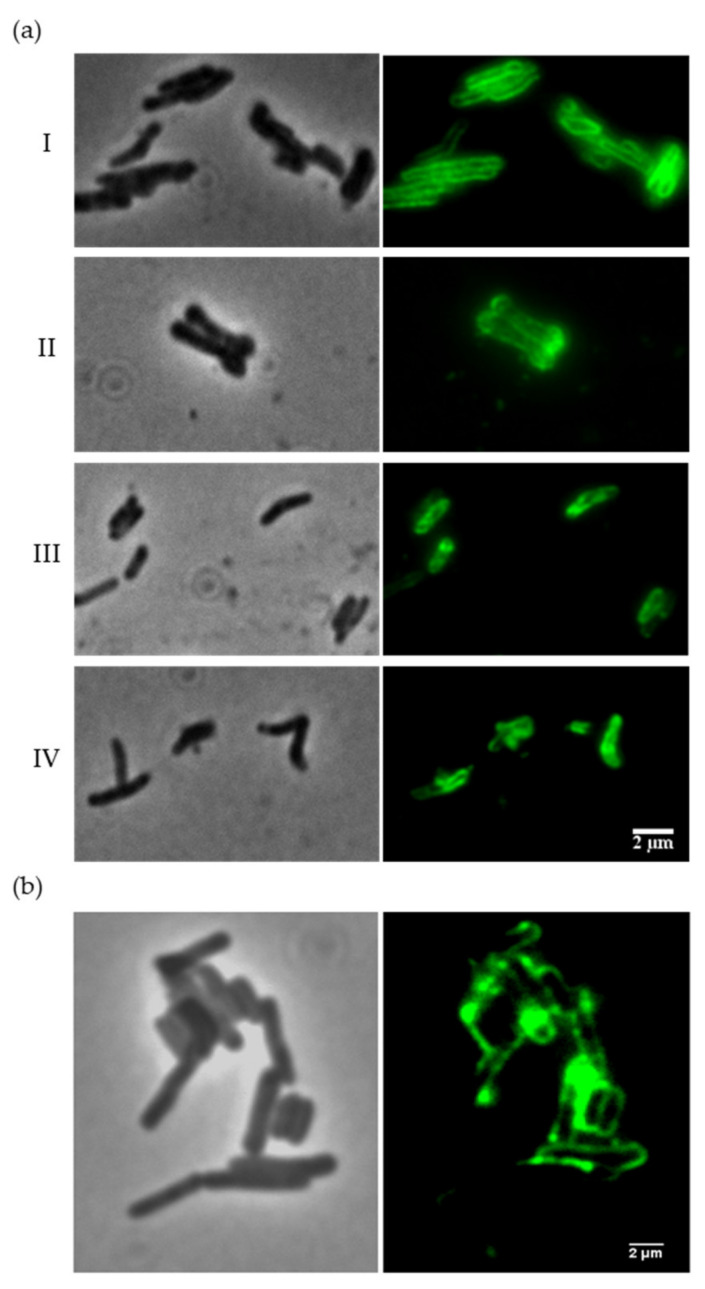
Cell-binding capacity of Ms6LysBPGBD-EGFP. (**a**) *M. smegmatis* (I), *M. vaccae* (II), *M. bovis* BCG (III) and *M. tuberculosis* H37Ra (IV) were treated with 1% SDS prior to incubation with the fusion protein. (**b**) *M. smegmatis* cells pretreated with the full-length Ms6 LysB. Representative images of each strain are shown in phase-contrast (**left**) and fluorescence microscopy (**right**). Exposure time of 100 ms; scale bar 2 µm.

To evaluate the binding capacity of the Ms6 LysB *N*-terminal region, we first constructed a recombinant protein by fusing the region encompassing amino acids 1–90 of Ms6 LysB to the Enhanced Green Fluorescence Protein (EGFP) and tested the ability of this protein (Ms6LysBPGBD-EGFP) to bind to mycobacteria cells. Since in mycobacteria the PG is not exposed due to the presence of an outer membrane, in an attempt to reach the PG, intact cells were first treated with 1% SDS for 1 h, washed with PBS and subsequently incubated with Ms6LysBPGBD-EGFP or EGFP (negative control). Due to the lipid nature of the mycobacterial outer membrane, treatment with detergents disturbs the membrane structure and apparently dissolves extractable lipids, which results in significant changes in permeability [14]. Mycobacteria have previously been shown to survive high concentrations of SDS [45,46]. Observation of SDS-treated cells by fluorescence microscopy clearly showed that the Ms6LysBPGBD-EGFP labels the cell surface of *M. smegmatis*, *M. vaccae*, *M. bovis* BCG and *M. tuberculosis* H37Ra (Figure 2a), resulting in a bright fluorescence of the entire cell surface, whereas EGFP alone did not label the cells (Figure S1). In mycobacteria cells without the SDS pre-treatment, no fluorescence was observed with the fusion protein (Figure S2), indicating that the target is not surface exposed.

Since Ms6 LysB hydrolyzes mycolic acids-containing lipids and the main target is the link between the OM and the PG-AG, in an attempt to detach the mycobacteria outer membrane, we also performed an experiment where we pretreated *M. smegmatis* cells with the full-length Ms6 LysB protein. Prior to the incubation with the fluorescent proteins, *M. smegmatis* cells were incubated with Ms6 LysB for 45 min (see methods for details). Again, the Ms6LysBPGBD-EGFP was able to bind *M. smegmatis* cells (Figure 2b), while no fluorescence was observed with the control EGFP (data not shown). These data indicate that the *N*-terminus region does indeed have a role in the binding of Ms6 LysB to the mycobacterial cell envelope. Importantly, our data also show the ability of Ms6 LysB to disturb the mycobacteria surface, allowing the access of the fusion protein to its target.

### 3.3. Ms6 LysB N-Terminus Binds to Peptidoglycan

**Figure 3:**
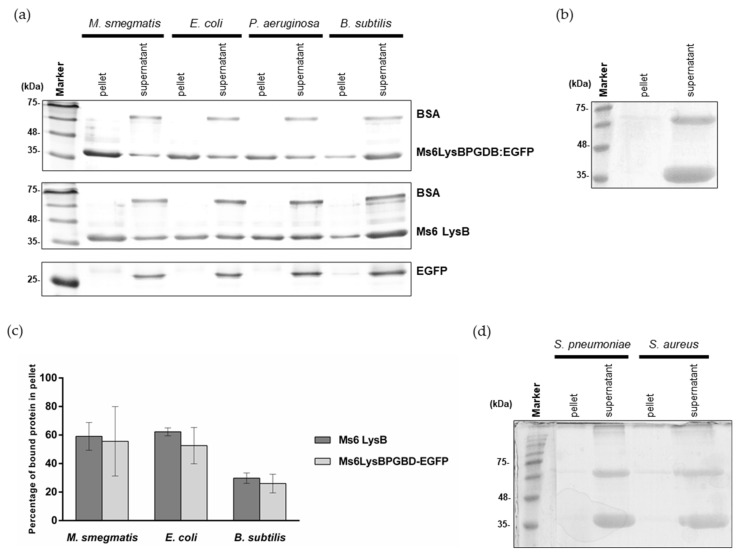
Pulldown assays of full-length Ms6 LysB and Ms6LysBPGBD-EGFP and EGFP with purified peptidoglycan from: (**a**) *M. smegmatis*, *E. coli*, *P. aeruginosa* and *B. subtilis.* Pellet and supernatant fractions were analyzed by SDS-PAGE followed by Comassie-blue staining. Bovine serum albumin (BSA) was used as a control and was only present in the supernatant fractions. (**b**) As a negative control, Ms6 LysB was incubated without peptidoglycan. (**c**) Quantification of pulldown assays. Quantification was performed with 3 independent gels, including the represented in Panel A. Ms6 LysB (dark grey), Ms6 LysBPGBD-EGFP (ligth grey). For each bar, the mean ± SD of three independent assays is indicated. (**d**) Pulldown assays of full length Ms6 LysB with purified peptidoglycan from *S. pneumoniae* and *S. aureus*. Again, BSA was used as a control and is only present in the supernatant fractions.

To specify that PG is the substrate, we evaluated the binding capacity of Ms6 LysB *N*-terminal region in a pulldown assay using purified PG from *M smegmatis*. 90 μg of Ms6LysBPGBD-EGFP were added to the pure PG and incubated for 45 min at room temperature. After centrifugation and washing steps, the pellet and supernatant fractions were analyzed by SDS-PAGE. In this assay, proteins that bind to the insoluble PG will co-precipitate with PG and will be found in the pellet fraction. Comparing the amounts of protein in each fraction, it is clear that Ms6LysBPGBD-EGFP was pulled down by PG (Figure 3a). In this experiment, BSA, a protein that does not bind to PG, was added to the reaction mix as a control for unspecific binding, to ensure that the PG pellet was adequately washed and that no unbound protein remained in the pellet fraction. As expected, the BSA band was only detected in the supernatant fraction indicating that only proteins that have ability to bind PG were in the pellet. To rule out the possibility of a nonspecific binding, we also performed a parallel assay with EGFP, where the PGBD is not present. As expected, the EGFP protein was only detected in the supernatant fraction (Figure 3a). The same ability to be pulled down by PG was observed with the full-length Ms6 LysB (Figure 3a), where the PGBD is present, strengthen the idea that the *N*-terminus of Ms6 LysB has peptidoglycan binding capacity.

In addition, to ensure that the presence of Ms6 LysB in the pellet fraction was not a result of protein precipitation, a negative control without PG was performed in the same conditions. In this case, the fusion protein was totally in the supernatant fraction (Figure 3b).

To determine the specificity of PG binding we also performed pulldown assays under exactly the same conditions as those described above, using PG from Gram-positive *B. subtilis*, *S. aureus and S. pneumoniae* and from Gram-negative *E. coli and P. aeruginosa.* As observed in Figure 3a, Ms6LysBPGBD-EGFP or Ms6 LysB were able to bind to the PG of *E. coli*, *P. aeruginosa* and *B. subtilis*, although each PG pulled down different amounts of protein, which suggested that the binding affinity was not the same for all the PGs tested. To assert this, the binding of both proteins to *M. smegmatis*, *E. coli* and *B. subtilis* PG was quantified. Ms6LysBPGBD-EGFP and Ms6 LysB showed similar ability to bind PG, with both proteins showing higher affinity for *M. smegmatis and E. coli* PGs. 59% and 62% of Ms6 LysB was bound to *M. smegmatis* and *E. coli* PG, respectively, while the binding to *B. subtilis* PG was of just 30%. Ms6LysBPGBD-EGFP showed a parallel PG binding profile, with binding to *B. subtilis* PG (26%) reaching about half of the observed for the PGs of *M. smegmatis* (56%) or *E. coli* (53%) (Figure 3c). No binding was detected for the PG of *S. aureus* or *S. pneumoniae* (Figure 3d). Of note is the fact that the binding capacity was observed in bacteria species belonging to the same PG chemotype. PG of *M. smegmatis*, *E. coli* and *B. subtillis* belong to A1γ chemotype (a 4→3 direct cross-linkage and *meso*-2,6-diaminopimelic acid at the third position of the peptide stem) whereas *S. aureus* and *S. pneumoniae* belong to chemotype A3γ (cross-linkage by interpeptide bridges and L-Lysin at the third position) [47,48]. These results suggest that a direct cross linkage and/or the presence of meso-DAP is determinant for Ms6 LysB binding. Although the PGs that pulled down LysB belong to the same chemotype, their glycan strands present different modifications. *B. subtillis* strains contain *N*-deacetylated Glc*N*Ac and Mur*N*Ac residues which may account for a reduced binding as a fully *N*-acetylated peptidoglycan is required for the binding of the bacteriophages φKZ and EL endolysins [40]. *N*-glycolylation of muramic acid is one of the hallmarks of mycobacteria; however, the role of the glycolyl residue is unknown, and how this glycan modifications influences the binding affinity of Ms6 LysB remains to be determined.

These results clearly demonstrate that the Ms6 LysB binding to the PG is dependent on the presence of the PGBD.

### 3.4. Deletion of PGBD from LysB Results in a Faster Rise Period

**Figure 4:**
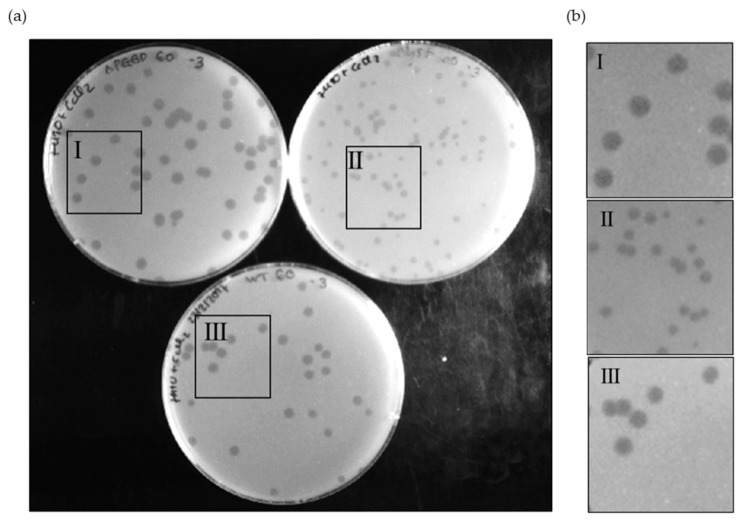
Phage plaque morphology of Ms6*lysB*ΔPGBD (I), Ms6Δ*lysB* (II) and Ms6*wt* (III) on a lawn of *M. smegmatis* (**a**), and 2× zoom of each highlighted area (**b**). The mutant Ms6*lysB*ΔPGBD forms slightly larger plaques than the Ms6*wt*, while Ms6Δ*lysB*, in turn, forms the smallest plaques. The pictures were taken at 24 h post infection.

Since the Ms6 LysB PGBD region is separated from the catalytic domain, we investigated the contribution of this *N*-terminal region to the phage lysis phenotype. Taking advantage of the Bacteriophage Recombineering of Electroporated DNA (BRED) technology [34], we constructed an Ms6 mutant (Ms6*lysB*ΔPGBD) lacking the sequence coding for this region (amino acids 1–90). This mutant phage produced phage plaques on *M. smegmatis* lawn with similar morphology to that of the Ms6*wt*, in contrast with Ms6Δ*lysB*, a mutant that does not produce LysB and that forms plaques with a reduced size [21] (Figure 4).

**Figure 5:**
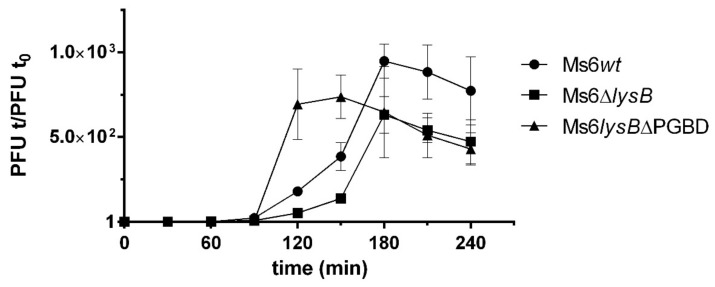
One-step growth curves of Ms6*lysB*ΔPGBD (triangles), Ms6*wt* (circles) or Ms6Δ*lysB* (squares) on *M. smegmatis*. Ms6ΔPGBD have a fast increase in the number of plaque-forming units (PFU) when compared to a Ms6*wt* or Ms6Δ*lysB* infection. Both curves show similar progression up to 90 min post adsorption showing no differences in the timing of lysis. T_0_ marks the end of the adsorption and start of the one-step experiment. The PFU/mL at t = 0 was used to normalize PFU/mL of each time point. For each time point, the mean ± SD of three independent assays is indicated.

To understand the importance of the LysB PGBD to phage lysis, one-step growth experiments were performed with Ms6*wt*, Ms6*lysB*ΔPGBD and Ms6Δ*lysB*. *M. smegmatis* cells were infected with Ms6*wt*, Ms6*lysB*ΔPGBD or Ms6Δ*lysB* at a multiplicity of infection (MOI) of one. All phages showed a latent period of 140 min, consisting of the initial 50 min of adsorption and an additional 90 min as shown in Figure 5, indicating that no changes in the timing of lysis occur. However, the rise period observed with Ms6*lysB*ΔPGBD was shorter than that of Ms6*wt* or Ms6Δ*lysB*. Both Ms6*wt* and Ms6Δ*lysB* showed a slow rise period of 90 min, while Ms6*lysB*ΔPGBD presented a rise period of 60 min, i.e., the maximum number of released phage particles was achieved at 150 min post-adsorption, in contrast with 180 min for the Ms6*wt* or Ms6Δ*lysB*.

## 4. Discussion

Bacteriophages are the most abundant biological entities on Earth, playing key roles in the biology of bacteria. For their own survival, they have to maintain a close interaction with their hosts, which for the most frequently isolated phages from nature (the tailed bacteriophages) leads to bacterial lysis. This is an important characteristic of bacteriophages, and is being explored in the development of anti-bacterials to fight pathogenic bacteria, particularly antibiotic-resistant strains. It is thus of huge importance to understand the mechanism of phage-induced lysis. To be released at the end of a lytic cycle, phages must overcome the cell envelope barriers. It was generally thought that degradation of the PG was sufficient for cell burst, allowing the spread of the newly synthesized phage particles to the environment. Although this is true for phages infecting Gram-positive bacteria, it has recently become clear that in Gram-negative hosts the OM is an important barrier and that its disruption is required for phage lysis. This function is achieved by a third class of lysis proteins, the spanins [3]. 

The studies performed by Ry Young and collaborators made a huge contribution to the comprehension of the role of each phage lysis protein in disruption of the bacterial cell envelope. It is now clear that each barrier must be sequentially eliminated, beginning with permeabilization of the inner membrane by the holins, followed by breaking of the peptidoglycan mesh by the endolysins and in Gram-negative hosts, the last barrier, the OM, is disrupted by the spanins [2,3,4].

In mycobacteriophages, the players in mycobacteria lysis have already been identified [49]. Distinct from other phage lysis cassettes is the presence of gene *lysB*, which encodes a lipolytic enzyme with mycolyl-arabinogalactan esterase activity and whose function is elimination of the lipid rich OM of mycobacteria [18,19].

In this work, we show that the mycobacteriophage Ms6 LysB has a modular architecture comprising an *N*-terminal region with structural similarity to the *N*-terminal peptidoglycan binding domain of phage φKZ endolysin and a predicted *C*-terminal catalytic module with structural similarity to the LysB protein of mycobacteriophage D29. Here we experimentally demonstrate the peptidoglycan binding capacity of Ms6 LysB to PG. The PGBD either present on the full-length Ms6 LysB or in the fusion protein Ms6LysBPGBD-EGFP allows the binding to the PG of *P. aeruginosa*, *E. coli*, *B. subtillis* and *M. smegmatis*, all sharing a A1γ peptidoglycan chemotype, suggesting a common feature for LysB PGBD targeting. However, our data suggest that the binding affinity is not identical in all tested bacteria, with *M. smegmatis* and *E. coli* PGs showing the highest percentages of binding and a slightly higher binding of Ms6LysBPGBD-EGFP to *M. smegmatis*. The glycan strands of the bacterial peptidoglycan, made of alternating β-1,4-linked Glc*N*Ac and Mur*N*Ac residues, are invariably modified or linked to other cell wall polymers. The biological role of such modifications (deacetylation, *O*-acetylation and *N*-glycolylation) may vary according to the type of modification. Deacetylated sugars in the PG strands as well as *O*-acetylated PG, present an increased resistance to the activity of lysozyme, a muramidase that cleaves the β-1,4-glycosidic linkage between Mur*N*Ac and Glc*N*Ac [50]. This decrease in activity was correlated with a decrease in substrate binding [48,51]. This suggests that chemical modifications of the PG from the different bacteria used in this study may account for the observed differences in the binding of Ms6 LysB and Ms6LysBPGBD-EGFP. Furthermore, Mur*N*Ac *N*-glycolylation, a modification characteristic of the *Mycobacterium* genera, has also been suggested to have a role in protection against lysozyme [52,53]. Although the role of the chemical modifications to PG binding was not investigated in this work, it is reasonable to hypothesize that modifications on the glycan strands may account for the observed differences in the binding of Ms6 LysB and Ms6LysBPGBD-EGFP. Nevertheless, our results clearly demonstrate that the *N*-terminus of Ms6 LysB is responsible for the binding of the protein to PG. Ms6 LysB is a late protein that acts in the last step of phage lysis. Similar to what happens with phage λ, the Ms6 holins, endolysins and LysB must also act sequentially with LysB disrupting the outer membrane (OM) of mycobacteria after disruption of the PG meshwork by the Ms6 endolysins, which exert their function after holin triggering. As mentioned before, λ spanins anchored to the CM and OM remain inactive until PG hydrolysis by the endolysin [3,7]. In parallel, it is reasonable to think that the Ms6 LysB *N*-terminus could serve to anchor the LysB, positioning it close to its target, keeping it inactive until disruption of the PG, which would release the protein and consequently cleave the link to the OM. This means that LysB would be positioned in the extracytoplasmic environment before holin is triggered. However, in contrast to spanins, no export signals were predicted for the Ms6 LysB, and how it accesses its target is still unknown.

The question that arises is what is the advantage for the phage fitness of LysB binding to PG, since deletion of the PGBD results in a one-step curve with a shorter rise period, meaning that the binding to the PG delays the full release of the viral progeny. Once lysis begins, the mutant phage release is more abrupt than in wild-type infection or when the full-length LysB is absent (Figure 5). Many Ms6 LysB homologues encoded in mycobacteriophage genomes do not contain this *N*-terminal region, as exemplified by the D29 LysB (Figure 1). Interestingly, a phylogenetic tree generated with CLUSTALW [54], with representatives of LysB phamilies (Phams) from different mycobacteriophage clusters, shows that an extended *N*-terminus is present in both close and distant branches of the tree, and 14 out of 51 contained a predicted peptidoglycan binding motif (Figure S3). Comparison of the biological parameters of phages encoding LysB with and without the *N*-terminal regions would give additional clues on the importance of this region to the lysis phenotype. In addition, how Ms6 LysB and homologues lacking this PGBD reach their target is a matter that deserves future investigation.

Of note is the fact that Ms6 LysB proved to disturb the mycobacteria cell envelope when added externally, as treatment of intact cells with the purified protein parallels the effect of SDS, allowing the binding of the fusion protein Ms6LysBPGBD-EGFP. This is consistent with the fact that Ms6 LysB, in addition to functioning as a mycolyl-arabinogalactan esterase, is also able to hydrolyze lipids present in the mycobacteria outer layer [19] which would disturb the OM, and somehow allow the binding to PG. This means that LysB proteins may be explored as tools to disturb the mycobacteria OM.

To our knowledge, this is the first work demonstrating the ability of a mycobacteriophage lysis protein LysB to bind to peptidoglycan.

## Figures and Tables

**Table 1:** Bacteria, bacteriophages and plasmids used in this work.

Bacteria, Plasmids and Bacteriophages	Description	Source and Reference
Bacteria		
*E. coli* JM109	*recA1 endA1 gyr96 thi hsdR17 supE44 relA1* Δ*(lac-proAB) [F’ traD36 proAB lacI^q^Z*ΔM15]	Stratagene
*Bacillus subtillis* MB24	*trpC*2 *metC*3	Laboratory stock
*Staphylococcus aureus* RN4220	Restriction-deficient derivative of *S. aureus* NCTC8325-4 strain capable of receiving foreign DNA	[24]
*Streptococcus pneumoniae Pen6*	R6Hex transformant with chromosomal DNA from penicillin resistant clinical isolate 8249 and selected for Pen^R^	[25]
*Pseudomonas aeruginosa*	ATCC 27853	American type culture collection
*Mycobacterium smegmatis* mc^2^155	High-transformation-efficiency mutant of *M. smegmatis* ATCC 607	[26]
*M. smegmatis* mc^2^155 (pJV53)	Recombinant strain containing plasmid pJV53 which expresses recombineering functions	[23]
*Mycobacterium tuberculosis* H37Ra	ATCC 25177	American type culture collection
*Mycobacterium vaccae*	SN 901 (IPP)	Institut Pasteur Production
*Mycobacterium bovis* BCG (Pasteur)	ATCC 35734	American type culture collection
Plasmids		
pQE30	Expression vector; T5 promoter; Amp^r^	Qiagen
pMP302	Ms6 *lysB* cloned into pQE30	[19]
pEGFP-N1	Vector containing the EGFP coding sequence	Clontech Laboratories
pQE30:*egfp*	*egfp* fragment cloned into pQE30	This study
pQE30:PGBD-*egfp*	Ms6 LysB PGBD coding sequence fused to *egfp* inserted into pQE30	This study
Bacteriophages		
Ms6	Temperate bacteriophage from *M. smegmatis*.	[27]
Ms6l*ysB*∆PGBD	Ms6 derivative mutant lacking the PGBD coding sequence	This study
Ms6∆l*ysB*	996 bp in-frame deletion of the Ms6 *lysB* gene	[21]

## Supplementary Materials

The following are available online at https://www.mdpi.com/article/10.3390/v13071377/s1, Table S1: Oligonucleotides used in this work; Figure S1: Negative control of EGFP cell binding capacity; Figure S2: Cell binding capacity of Ms6LysBPGBD-EGFP to untreated *Mycobacterium smegmatis* mc^2^155; Figure S3: Phylogenetic tree of 51 mycobacteriophage LysB proteins.
