# Supplementary material for: The Mycobacteriophage Ms6 LysB N-Terminus Displays Peptidoglycan Binding Affinity
Source: Viruses. 2021 Jul 15;13(7):1377. doi: 10.3390/v13071377 (PMC8309991; doi:10.3390/v13071377)
Supplement: Supplementary file 1 [file Figure S3.pdf › Supplementary Material/Figure S3.pdf]

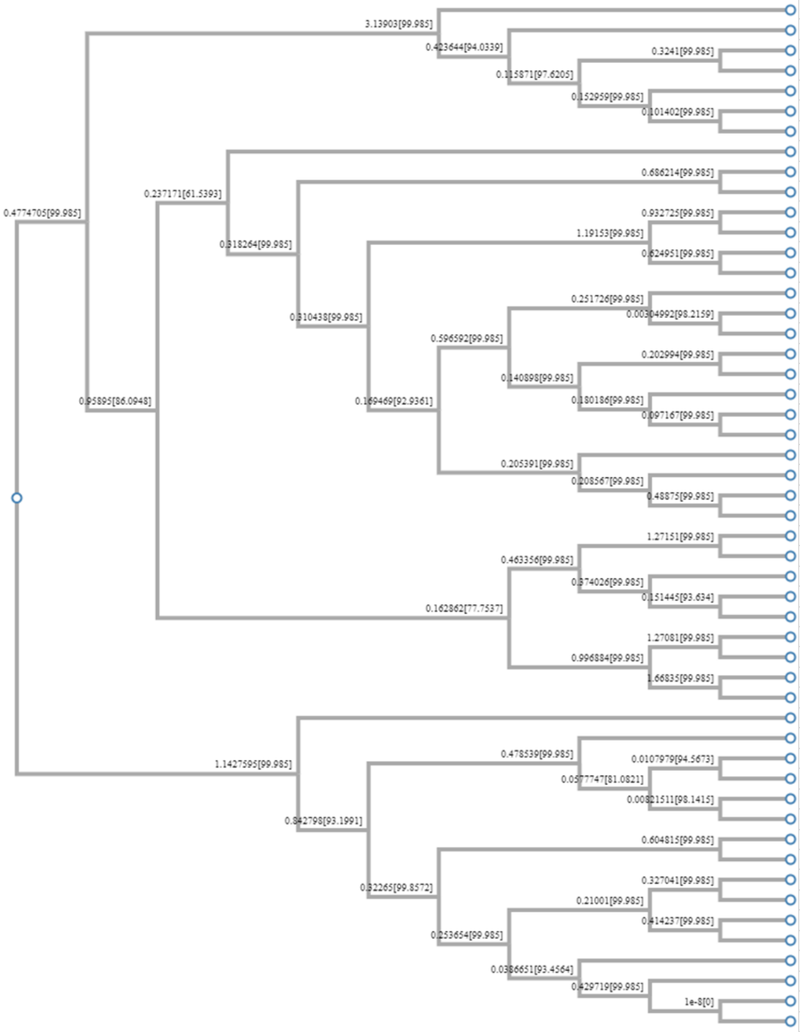

Phage	Cluster	Gene	Pham	N-terminus length (aa)	Putative PG binding domain
Apex	B	45	66684	106	Yes
Avocado	G	27	66684	85	Yes
Che9c	I	26	66684	92	Yes
Phayonce	P	27	66684	91	Yes
FF47	AB	32	66684	81	Yes
Adjutor	D	40	66684	89	Yes
Konstantine	H	43	66684	84	Yes
Superphikiman	J	48	66848	78	No
GardenSalsa	M	43	66848	76	Yes
Bongo	M	38	66848	75	Yes
ChaChing	B	47	66848	96	No
Abinghost	B	47	66848	94	No
Candle	R	39	66848	93	Yes
Papyrus	R	39	66848	93	Yes
Salacia	C	254	66848	95	No
Bxz1	C	238	66848	95	Yes
Pio	C	259	66848	95	No
Vorrps	O	67	66848	98	No
Corndog	O	70	66848	98	No
Leperchaun	F	31	66848	91	Yes
Fruitloop	F	30	66848	89	No
Ms6	-	25	-	90	No
Cosmo	V	52	66848	83	Yes
D29	A	12	66848	-	-
Acquire49	L	26	66848	93	No
Gabriela	L	28	66848	93	No
Miramae	A	10	66848	73	No
Nyxis	A	10	66848	74	No
Adephagia	K	31	66848	-	-
Hurricane	K	32	66848	-	-
Eponine	K	34	66848	-	-
Omega	J	53	66848	-	-
Constella	J	41	66848	-	-
Porky	E	35	66848	-	-
Bruin	E	35	66848	-	-
MrMiyagi	AC	34	66848	-	-
Babsiella	I	27	66783	86	No
Brujita	I	30	66783	82	No
Butters	N	27	66783	82	No
Majeke	P	27	66783	82	No
Phineas	P	27	66783	82	No
Skippy	B	49	66783	103	No
Orion	B	50	66783	103	No
Hail	Q	32	66783	103	No
Luke	Q	33	66783	103	No
Tiger	A	8	66783	-	-
Cuco	A	8	66783	-	-
Typha	Y	32	66783	99	No
Angel	G	28	66783	96	No
BPs	G	28	66783	96	No
Phreak	N	28	66783	96	No

Figure S3. Phylogenetic tree of 51 mycobacteriophage LysB proteins with branch length depicted. The cluster of the phages, the gene coding for the LysB and the corresponding pham (available on The Actinobacteriophage Database [17]) were included. Phage cluster and LysB pham are not shown for Ms6 since it is not included in this database. Length of the extra N-terminal region was accessed by HHpred for all proteins. When existant, MotifFinder tool was used to verify the presence of the putative peptidoglycan binding domain in this region. The symbol (-) denotes absence of the N-terminal region.
