# Supplementary material for: The Mycobacteriophage Ms6 LysB N-Terminus Displays Peptidoglycan Binding Affinity
Source: Viruses. 2021 Jul 15;13(7):1377. doi: 10.3390/v13071377 (PMC8309991; doi:10.3390/v13071377)
Supplement: Supplementary file 1 [file viruses-13-01377-s001.zip › Supplementary Material/Table S1.pdf]

Table S1. Oligonucleotides used in this work.

Name	Sequence[a]	Comments
PrPGBDFw	GCGGATCCCGCATCGACGGCGAATAC	Includes BamHI site to clone in pQE30
PrPGBDRv	GCGGTACCGTCGATGACGGGCCGGGA	Includes KpnI site to clone in pQE30
PrEGFPaFw	GCGGTACCGTGAGCAAGGGCGAGGAG	Includes KpnI site to clone *egfp* in pQE30 and to generate fusions in pQE30
Pr EGFP Rv	GCAAGCTTCTTGTACAGCTCGTCCATGCC	Includes HindIII site to clone in pQE30
lysBΔPGBD	GGCGGAAAAACCCTCGTGGACGCGGTAGCAGAACTGTTGGGCCACTGATGACCCGGCCAGTCCTGTTCACCGTGTGCGGCACCGGCGTGCCCTGGTGGGT	100bp oligonucleotide to generate Ms6*lysB*ΔPGBD
PrExtΔlysBFw	AGATCCTGCGGCAACTGCGCGGATACAACCTCACTGGCTGGCCGCAGCTCGGCGGAAAAACCCTCGTGGACGCGG	Extend *lysB*ΔPGBD
PrExtlysBΔPGBD Rv	CGAGATCCTGCGGCAACTGCGCGGATACAACCTCACTGGCTGGCCGCAGCTCGGCGGAAAAACCCTCGTGGACG	*lysB*ΔPGBD
PrlysA120Fw	GCGAGCTCAAGTCACGCATCGCCACCGTC	Flanking primer for deletion screening
PrlysBendRv	GCATGGGTACCGCCTCCTATGTGC	Flanking primer for deletion screening

[a] Restriction enzymes sites are underlined.
