# Supplementary material for: The Mycobacteriophage Ms6 LysB N-Terminus Displays Peptidoglycan Binding Affinity
Source: Viruses. 2021 Jul 15;13(7):1377. doi: 10.3390/v13071377 (PMC8309991; doi:10.3390/v13071377)
Supplement: Supplementary file 1 [file viruses-13-01377-s001.zip › Supplementary Material/Figure S1.pdf]

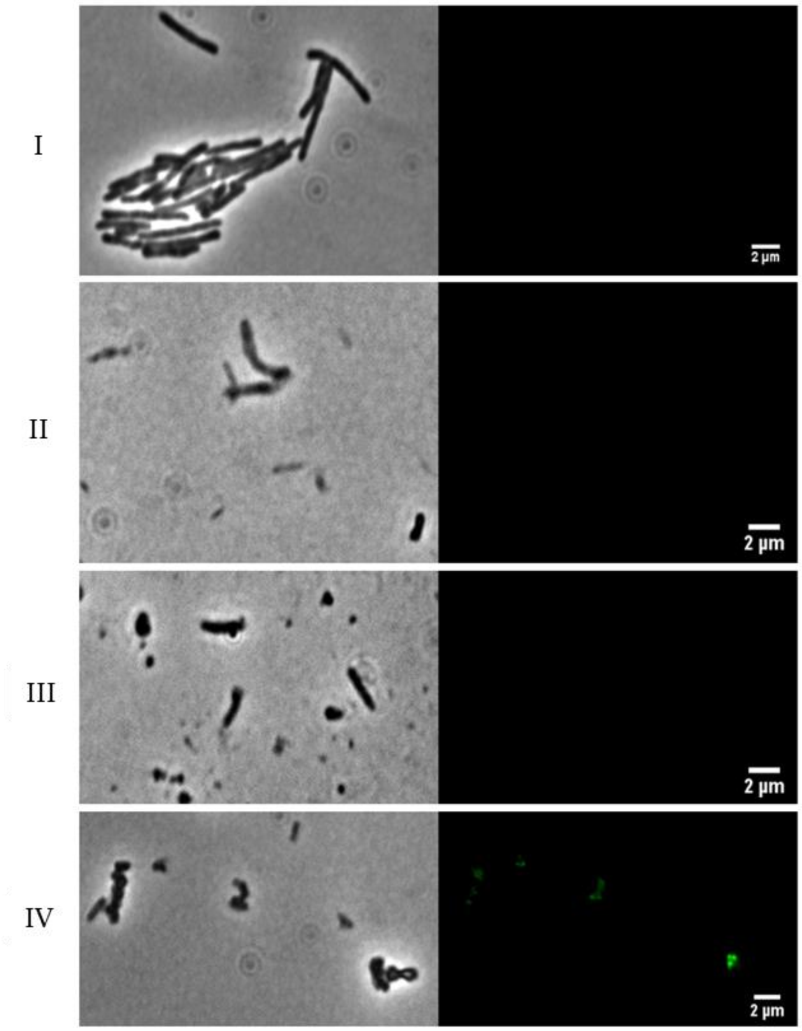

Figure S1. Negative control of EGFP cell binding capacity. (I) *Mycobacterium smegmatis;* (II) *Mycobacterium vaccae*; (III) *Mycobacterium bovis BCG*; (IV) *Mycobacterium tuberculosis* H37Ra. Representative images of each strain are shown in phase-contrast (left) and fluorescence microscopy (right) . Exposure time of 100 ms; scale bar of 2 μm indicated for each montage.
