# Supplementary material for: The Mycobacteriophage Ms6 LysB N-Terminus Displays Peptidoglycan Binding Affinity
Source: Viruses. 2021 Jul 15;13(7):1377. doi: 10.3390/v13071377 (PMC8309991; doi:10.3390/v13071377)
Supplement: Supplementary file 1 [file viruses-13-01377-s001.zip › Supplementary Material/Figure S2.pdf]

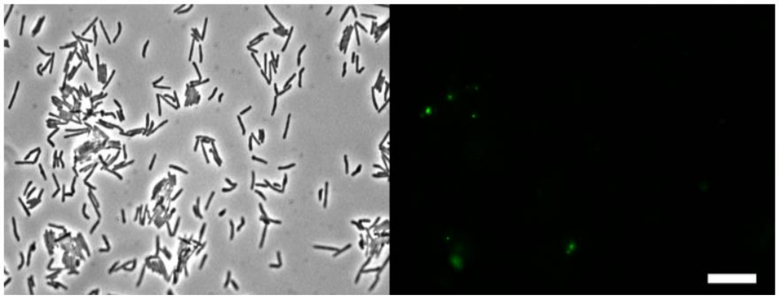

Figure S2. Cell binding capacity of Ms6LysBPGBD-EGFP to untreated *Mycobacterium smegmatis* mc²155. Phase-contrast (left) and fluorescence microscopy (right). Exposure time of 100 ms; scale bar of 10 μm.
